# *mBio* Welcomes Clinical Research Papers That Advance Our Understanding of Human-Microbe Interactions

**DOI:** 10.1128/mbio.00527-22

**Published:** 2022-03-29

**Authors:** Arturo Casadevall, Robert A. Bonomo, Martin J. Blaser, Samuel Miller, Liise-anne Pirofski

**Affiliations:** a W. Harry Feinstone Department of Molecular Microbiology and Immunology, Johns Hopkins Bloomberg School of Public Health, Baltimore, Maryland, USA; b Louis Stokes Veterans Affairs Medical Center, Cleveland, Ohio, USA; c Rutgers University, Rutgers, New Jersey, USA; d University of Washington, Seattle, Washington, USA; e Albert Einstein College of Medicine, Bronx, New York, USA

**Keywords:** clinical, *mBio*, scope

## EDITORIAL

The COVID-19 pandemic has brought great suffering to millions and unsettled many of the structures of everyday life and in doing so provided a powerful reminder that epidemic infectious diseases can threaten humanity and our civilization. The COVID-19 pandemic also provides clear evidence that science can protect society and alleviate suffering by delivering new vaccines, antivirals, antibody-based therapies, rapid diagnostics, and amazingly detailed epidemiological information through the power of new OMIC technologies. These advances were developed in “real time” and deployed rapidly. The COVID-19 pandemic also reminded us how a crisis in medicine illuminates gaps in our scientific knowledge that trigger new research directions. Hence, to address the clinical and scientific awakening brought about by the pandemic, *mBio* is expanding its scope to welcome clinical papers that address the nature and outcomes of human-microbe interactions and microbial diseases and early novel intervention strategies.

*mBio* is already a journal with a broad scope that includes reports of clinical findings that address microbial pathogenesis and diseases. Until now, *mBio* has largely focused on publishing “cutting-edge” preclinical research. These studies tend to address human health and use samples from patients rather than report the results of an intervention or clinical observation. This strategy was extremely successful and led the journal to publish many new insights into pathogenesis and immunology. Although observational and interventional clinical studies would have been considered in the past had they been submitted to *mBio*, our journal has not previously sought them.

With this editorial, signed by five current editors who are physician-scientists, we issue a call for clinical papers that advance our understanding of human-microbe interactions, microbial diseases, immune responses, new therapies, and diagnostics. In recent decades, in our opinion, clinical research has become increasingly singular in its focus on disease outcomes, deemphasizing clinical scientific observation, which can be an important pipeline to insights that can fuel hypothesis testing. Many major clinical journals do not consider studies that explore, advance, or deviate from the confines of the study design and their prespecified outcomes, even though discoveries stemming from heterogeneity or unexpected diversity often lead to unexpected insights and, ultimately, to improved care. For example, many journals prohibit reporting subgroup analyses when a primary outcome is not met to avoid “cherry-picking” data even when such analyses are biologically plausible and clinically important. Presently, the evolution of randomized clinical trials, which remain the gold standard for evidence in clinical science, has been characterized by increasing rigidity (1). These trends coincide with the rise of corporate medicine and the need for simplified algorithms to improve patient flow, leaving many in the medical community wishing for more.

*mBio* is interested in human studies in which the intervention or observation revealed a new facet of human-microbe relationships or of the characteristics of the infecting microbe or the immune response. As a result of this novel focus, case reports or papers focused solely on therapeutic interventions that do not advance our basic scientific understanding of host-microbe interaction and/or the human immune response are discouraged. All relevant clinical research will be rigorously reviewed to ensure that it complies with ethical and regulatory guidelines and has sound statistical analysis. We look forward to evaluating your submissions and opening this new chapter in clinical investigations of infectious diseases. Lastly, we reassure our readers, authors, and the entire *mBio* community that this initiative is an additional focus for the journal, not a refocusing of the journal toward clinical sciences. *mBio* will continue to welcome and publish within the broad scope of microbial-related science that it has championed for the past decade.
